# Periodontitis is associated with stroke

**DOI:** 10.1186/s12967-023-04545-1

**Published:** 2023-10-06

**Authors:** Xiaodan Zheng, Xin Li, Juanying Zhen, Dai Xue, Jun Hu, Qing Cao, Aimin Xu, Bernard Man Yung Cheung, Jun Wu, Chao Li

**Affiliations:** 1 Department of Neurology, Peking University Shenzhen Hospital, 1120 Lianhua Rd, Futian District, Shenzhen, 518036 Guangdong China; 2 Department of Stomatology, Shenzhen University General Hospital, Shenzhen University Clinical Medical Academy, Shenzhen, Guangdong China; 3 Department of Neurology, Shenzhen Samii Medical Center (The Fourth People’s Hospital of Shenzhen), Shenzhen, Guangdong China; 4 Institute of Stomatological Research, Shenzhen University, Shenzhen, Guangdong China; 5 Department of Medicine, School of Clinical Medicine, The University of Hong Kong, Queen Mary Hospital, Pokfulam, Hong Kong SAR China; 6 Department of Stomatology, Affiliated Children’s Hospital of Jiangnan University, Wuxi, Jiangsu China; 7 Department of Otolaryngology, Affiliated Eye and ENT Hospital, Fudan University, Shanghai, China; 8 State Key Laboratory of Pharmaceutical Biotechnology, The University of Hong Kong, Pokfulam, Hong Kong SAR China

**Keywords:** Periodontitis, Stroke, Inflammation

## Abstract

**Background:**

Periodontitis is considered as a risk factor for cardiovascular diseases and atherosclerosis. However, the relationship between periodontitis and stroke is rarely studied. Therefore, we aimed to explore the relationship between periodontitis and stroke.

**Methods:**

Statistical analysis was performed using the complex sampling design. We analyzed data on 6,460 participants, representing 92,856,028 American citizens aged 30 years or older, who had valid data on periodontitis and stroke from the National Health and Nutrition Examination Survey 2009–2014. We used clinical attachment level and probing pocket depth precisely to determine periodontitis and it is the first time to use such a precise method for exploring the relationship between periodontitis and stroke.

**Results:**

39.9% of participants had periodontitis and 2.1% of participants had a record of stroke diagnosis. Stroke was associated with severity levels of periodontitis (p for trend = 0.018). The odds ratio for stroke was significantly elevated in the severe periodontitis and moderate periodontitis participants compared to participants without periodontitis (OR for severe periodontitis: 2.55, 95% CI 1.25–5.21; OR for moderate periodontitis: 1.71, 95% CI 1.17–2.50). After adjusting for race/ethnicity and sex, the association remained significant (p for trend = 0.009). After further adjusting for BMI, hypercholesterolemia, diabetes, alcohol consumption and physical activity, the association still existed (p for trend = 0.027). The association was significant consistently after further adjusting for age (p for trend = 0.033).

**Conclusions:**

In this nationally representative study, we found an association between periodontitis and stroke. The risk of stroke in participants with severe periodontitis and moderate periodontitis was 2.55 times and 1.71times as high as those without periodontitis. Dental health management may be of benefit to stroke prevention.

**Supplementary Information:**

The online version contains supplementary material available at 10.1186/s12967-023-04545-1.

## Introduction

Periodontitis contributes to the development of systemic diseases, including coronary heart disease and diabetes [1, 2]. A prospective cohort study showed that during follow-up from 2014 to 2019, the risk of coronary heart disease increased by 37% in participants with periodontitis compared to those without periodontitis at baseline [3]. It was reported that individuals with type 2 diabetes had the potential to improve glycemic control and C-reactive protein concentrations by periodontal intervention [4]. The main pathophysiological mechanism was related to release of inflammatory mediators from periodontal tissue or translocated circulating oral microbiota [5].

Stroke is one of the common cardiovascular diseases. Increasing evidences suggested that inflammation plays an important role in stroke development [6]. As a chronic inflammatory disease, periodontitis is characterized by tissue infection around the teeth, resulting from complex interplay of bacterial infection and host responses [7]. Nearly 50% of US adults aged 30 or older suffer from periodontitis [8, 9]. The relationship between periodontitis and stroke from previous studies is controversial. An investigation into the Atherosclerosis Risk in Communities Study showed that periodontal disease was significantly associated with cardioembolic and thrombotic stroke subtypes, and indicated that the two diseases are linked by common inflammatory pathways [10]. However, in the Women's Health Initiative Observational Study, 1 in 4 reported a history periodontitis diagnosis which was not associated with elevated risks of cardiovascular disease events or cardiovascular disease mortality [11]. Currently there are a lack of studies based on both large population and precise periodontitis degrees in literature that have examined the relationship between periodontitis and stroke. Therefore, our study attempted to investigate the relationship between these diseases in US adults by using data from the 2009–2014 National Health and Nutrition Examination Survey (NHANES).

## Methods

### Study population

Data was acquired from the NHANES 2009–2014 in this study. In the NHANES 2009–2014, full-mouth periodontal examination protocol, a gold standard to determine periodontal status, has been used to evaluate the periodontitis prevalence in the US adults. The Centers for Disease Control and Prevention Institutional Review Board gave its approval to the NHANES protocols. All participants agreed to use their information in the research and gave informed consent. By using stratified, multi-stage probability sampling designs, NHANES obtains a presentative sample of the civilian non-institutionalized US population. Data about demographics, socioeconomics, dietary and health-related questions were collected in the NHANES interview. Medical measurements, dental measurements, physiological measurements and laboratory tests, were conducted in mobile examination centers. The NHANES website provided details on methodology and guidelines (http://www.cdc.gov/nchs/nhanes.htm) [12].

In the NHANES 2009–2014, there were 7042 individuals aged 30 years or older with available data on stroke and periodontitis. Our study included 6460 participants who had available information on body mass index (BMI), diabetes, hypercholesterolemia, hypertension, smoking, physical activity and alcohol consumption. After survey-weighting of complex sampling design, these participants represented 92,856,028 American citizens (see Additional file 1: Fig. S1).

### Variables of interest

Periodontitis was the primary variable of interest in this study. The periodontal evaluation was available to survey participants aged 30 years or older who had at least one tooth, excluding third molars. Clinical attachment level (CAL) and probing pocket depth (PPD) were assessed by using periodontal probes to measure gingival recession and pocket depth at six sites on each tooth. Periodontal measurements were rounded to the lowest whole millimeter. Periodontitis was classified into mild periodontitis, moderate periodontitis and severe periodontitis according to the 2012 Centers for Disease Control and Prevention/American Academy of Periodontology case definitions [13, 14]. Participants who met both the following two criteria were considered to have mild periodontitis: (1) ≥ 2 interproximal sites with CAL ≥ 3 mm, (2) ≥ 2 interproximal sites with PPD ≥ 4 mm (not on the same tooth) or one site with PPD ≥ 5 mm. Participants were considered to have moderate periodontitis if they met one of the following two criteria: (1) ≥ 2 interproximal sites with CAL ≥ 4 mm (not on the same tooth), (2) ≥ 2 interproximal sites with PPD ≥ 5 mm (not on the same tooth). Participants who met both the following two criteria were considered to have severe periodontitis: (1) ≥ 2 interproximal sites with CAL ≥ 6 mm (not on the same tooth), (2) ≥ 1 interproximal site with PPD ≥ 5 mm (see Additional file, Additional file 1: Table S1) [13, 14].

Participants’ responses to the survey question ‘Has a doctor or other health professional ever told you/survey participant that you have/she/he has a stroke?’ were used to define the diagnosis of stroke.

Cofounders which could be related to either periodontitis or stroke are included in the regression models [15, 16]. Race/ethnicity was identified by responses to self-reported demographic questionnaires and categorized as non-Hispanic whites, non-Hispanic blacks, Mexican Americans and others. BMI, which was measured in kg/m^2^, was calculated by dividing weight by height square. The definition of hypertension was a previous diagnosis by a professional, taking anti-hypertensive medication, or average blood pressure ≥ 140/90 mmHg. An individual with a fasting glucose level ≥ 7.0 mmol/L (126 mg/dL), glycohemoglobin ≥ 6.5%, taking anti-diabetic medication, or previous doctors' diagnosis was considered to have diabetes. Diagnosis of hypercholesterolemia was based on total serum cholesterol ≥ 6.2 mmol/L (240 mg/dL), a previous medical diagnosis, or the use of hypercholesterolemia medication. If participants have vigorous or moderate activity, they were considered physically active. Moderate activities are activities for at least 10 min continuously that require moderate physical effort and cause small increases in breathing or heart rate, and vigorous activities are activities for at least 10 min continuously that require hard physical effort and cause large increases in breathing or heart rate [12]. Participants were considered to be smokers if they had smoked ≥ 100 cigarettes in their lifetime [17]. Alcohol consumption was regarded as consuming alcoholic beverages at least once a week, 5 times a month, or 53 times a year during the past 12 months [18].

### Statistical analysis

Statistical analysis was performed by using the complex sampling procedure in SPSS version 22.0 (IBM Corporation, Armonk, New York). Six-year survey weights were calculated and used in all analyses to adjust for unequal selection probability and non-response bias according to NHANES analytical guidelines. Different characteristics of participants were compared using variance analysis. In order to calculate the odds ratios and 95% confidence intervals for the relationship between the severity levels of periodontitis and stroke, we used the logistic regression models. Variables that were used for adjustment as covariates in the regression models included age, race/ethnicity, sex, BMI, diabetes, hypercholesterolemia, hypertension, alcohol consumption, smoking and physical activity. P values ≤ 0.05 were considered statistically significant.

## Results

We analyzed 6460 adults aged ≥ 30 years participating in NHANES 2009–2014, including 3123 participants who had periodontitis (39.9%) and 181 participants who had stroke (2.1%).

Table 1 lists all participants' demographic characteristics and other potential confounders according to the presence of stroke. There were significant association of stroke with age (p < 0.001) and race/ethnicity (p = 0.014). Participants suffering from stroke had a higher prevalence of smoking (p = 0.001) in comparison with participants without stroke. Diabetes (p < 0.001), hypertension (p < 0.001), and hypercholesterolemia (p = 0.001) were all significantly associated with stroke. Besides, significant differences in alcohol consumption (p < 0.001) and periodontitis (p = 0.018) were also observed between the participants with and without stroke. However, there was no significant relationship of stroke with BMI, sex and physical activity in our study.

**Table 1:** Characteristics of all 6460 participants with and without stroke

Characteristics	Stroke	No stroke	P value
N	181	6279	
Age, years	64.1(1.2)	51.1(0.3)	< 0.001
Men, %	47.6(5.9)	49.6(0.7)	0.749
BMI, kg/m^2^	30.1(0.8)	29.3(0.2)	0.226
Race/ethnicity, %			0.014
Mexican American	6.5(1.6)	8.0(1.3)	
Non-Hispanic White	62.7(4.6)	69.2(2.4)	
Non-Hispanic Black	16.5(3.3)	10.1(1.2)	
Others	14.3(3.8)	10.1(1.2)	
Diabetes, %	32.7(4.7)	13.1(0.6)	< 0.001
Hypercholesterolemia, %	63.0(4.4)	45.9(1.0)	0.001
Hypertension, %	81.8(4.9)	41.0(1.0)	< 0.001
SBP, mmHg	127.8(1.8)	122.4(0.4)	0.002
DBP, mmHg	68.4(1.2)	71.9(0.3)	0.001
Alcohol consumption, %	17.5(3.6)	38.9(1.4)	< 0.001
Smoking, %	61.9(4.6)	43.6(1.0)	0.001
Physical activity, %	33.8(4.1)	41.5(1.2)	0.073
Periodontitis, %			0.018
No Periodontitis	46.3(5.3)	60.4(1.7)	
Mild Periodontitis	0.5(0.5)	2.6(0.4)	
Moderate Periodontitis	38.7(3.8)	29.5(1.5)	
Severe Periodontitis	14.5(3.9)	7.4(0.6)	

Data are expressed as mean or percent (SE)

*BMI* body mass index; *DBP* diastolic blood pressure; *SBP* systolic blood pressure

Table 2 presents the characteristics of all participants grouped by severity of periodontitis. Severe periodontitis was associated with proportions of men (p < 0.001), hypertension (p < 0.001), alcohol consumption (p = 0.003), smoking (p < 0.001), physical activity (p = 0.007) and stroke (p = 0.018). A significant difference was discovered among racial/ethnic groups (p < 0.001). Moderate periodontitis was associated with age (p < 0.001) and diabetes (p < 0.001). However, BMI and hypercholesterolemia were not significantly associated with periodontitis in our study.

**Table 2:** Characteristics of all 6460 participants among different periodontitis severity levels

Characteristics	No periodontitis	Mild periodontitis	Moderate periodontitis	Severe periodontitis	P value
N	3337	143	2285	695	
Age, years	49.1(0.4)	47.6(1.6)	55.5(0.5)	54.3(0.6)	< 0.001
Men, %	43.5(1.1)	60.9(5.0)	55.0(1.1)	72.2(2.3)	< 0.001
BMI, kg/m^2^	29.0(0.2)	29.9(0.8)	29.8(0.3)	29.3(0.4)	0.057
Race/ethnicity, %					< 0.001
Mexican American	5.9(1.0)	8.9(2.5)	11.2(1.9)	11.4(2.2)	
Non-Hispanic White	75.1(2.0)	61.6(5.4)	61.2(3.1)	54.7(4.6)	
Non-Hispanic Black	7.5(0.8)	11.6(3.2)	12.8(1.7)	21.1(3.4)	
Others	11.5(0.9)	17.9(3.5)	14.8(1.3)	12.8(1.6)	
Diabetes, %	9.8(0.6)	13.5(2.7)	19.6(1.1)	19.3(2.0)	< 0.001
Hypercholesterolemia, %	44.8(1.3)	44.0(10.2)	50.2(1.6)	42.7(2.3)	0.063
Hypertension, %	36.8(1.1)	42.7(6.8)	48.8(1.5)	54.0(2.8)	< 0.001
SBP, mmHg	120.2(0.4)	121.3(2.5)	125.9(0.7)	128.3(1.2)	< 0.001
DBP, mmHg	72.0(0.4)	70.9(1.3)	71.2(0.4)	73.5(0.8)	0.088
Alcohol consumption, %	40.4(1.8)	35.7(1.3)	33.6(1.8)	42.8(3.0)	0.003
Smoking, %	36.3(1.3)	29.9(4.5)	55.2(1.2)	66.3(2.5)	< 0.001
Physical activity, %	39.6(1.4)	42.5(6.1)	43.1(1.5)	47.5(2.1)	0.007
Stroke, %	1.6(0.3)	0.4(0.4)	2.7(0.3)	4.0(1.1)	0.018

Data are expressed as mean or percent (SE)

BMI, body mass index; DBP, diastolic blood pressure; SBP, systolic blood pressure

Table 3 shows the association between periodontitis and stroke. The odds ratio for stroke was significantly elevated in the severe periodontitis and moderate periodontitis participants compared to participants without periodontitis (OR for severe periodontitis: 2.55, 95% CI 1.25–5.21; OR for moderate periodontitis: 1.71, 95% CI 1.17–2.50). Stroke was associated with severity levels of periodontitis (p for trend = 0.018). However, mild periodontitis was not associated with stroke in our study. After adjusting for race/ethnicity and sex, the association remained significant (p for trend = 0.009). Compared to participants without periodontitis, the adjusted odds ratio for stroke were greater in the participants with severe periodontitis (OR = 2.54, 95% CI 1.35–4.78) and moderate periodontitis (OR = 1.71, 95% CI 1.20–2.44). After further adjusting for BMI, hypercholesterolemia, diabetes, alcohol consumption and physical activity, the association still existed (p for trend = 0.027). Compared to participants without periodontitis, the adjusted odds ratio for stroke was elevated in the severe periodontitis participants (OR = 2.39, 95% CI 1.23–4.62).

**Table 3:** Association between severity of periodontitis and stroke (Model 3: further adjusted for age)

	Unadjusted model	model 1	model 2	model 3
No periodontitis				
OR (95%CI)	Ref.	Ref.	Ref.	Ref.
Mild periodontitis				
OR (95%CI)	0.23(0.03–1.61)	0.23(0.03–1.59)	0.20(0.03–1.50)	0.24(0.02–1.93)
Moderate periodontitis				
OR (95%CI)	1.71(1.17–2.50)	1.71(1.20–2.44)	1.41(0.98–2.05)	0.93(0.64–1.35)
Severe periodontitis				
OR (95%CI)	2.55(1.25–5.21)	2.54(1.35–4.78)	2.39(1.23–4.62)	1.83(0.94–3.59)
P value for trend	0.018	0.009	0.027	0.033

Model 1: adjusted for race/ethnicity and sex

Model 2: further adjusted for BMI, hypercholesterolemia, diabetes, alcohol consumption and physical activity

Model 3: further adjusted for age

We further adjusted for age, smoking and hypertension respectively in Tables 3, 4 and 5, which were shown in model 3. Stroke was associated with severity levels of periodontitis after further adjusting for age (p for trend = 0.033). The association was no longer significant after further adjustment for smoking. The association was also no longer significant after further adjustment for hypertension.

**Table 4:** Association between severity of periodontitis and stroke (Model 3: further adjusted for smoking)

	Unadjusted model	model 1	model 2	model 3
No periodontitis				
OR (95%CI)	Ref.	Ref.	Ref.	Ref.
Mild periodontitis				
OR (95%CI)	0.23(0.03–1.61)	0.23(0.03–1.59)	0.20(0.03–1.50)	0.21(0.03–1.58)
Moderate periodontitis				
OR (95%CI)	1.71(1.17–2.50)	1.71(1.20–2.44)	1.41(0.98–2.05)	1.22(0.83–1.81)
Severe periodontitis				
OR (95%CI)	2.55(1.25–5.21)	2.54(1.35–4.78)	2.39(1.23–4.62)	1.94(0.98–3.87)
P value for trend	0.018	0.009	0.027	0.096

Model 1: adjusted for race/ethnicity and sex

Model 2: further adjusted for BMI, hypercholesterolemia, diabetes, alcohol consumption and physical activity

Model 3: further adjusted for smoking

**Table 5:** Association between severity of periodontitis and stroke (Model 3: further adjusted for hypertension)

	Unadjusted model	model 1	model 2	model 3
No periodontitis				
OR (95%CI)	Ref.	Ref.	Ref.	Ref.
Mild periodontitis				
OR (95%CI)	0.23(0.03–1.61)	0.23(0.03–1.59)	0.20(0.03–1.50)	0.19(0.02–1.50)
Moderate periodontitis				
OR (95%CI)	1.71(1.17–2.50)	1.71(1.20–2.44)	1.41(0.98–2.05)	1.22(0.85–1.76)
Severe periodontitis				
OR (95%CI)	2.55(1.25–5.21)	2.54(1.35–4.78)	2.39(1.23–4.62)	1.98(1.07–3.66)
P value for trend	0.018	0.009	0.027	0.073

Model 1: adjusted for race/ethnicity and sex

Model 2: further adjusted for BMI, hypercholesterolemia, diabetes, alcohol consumption and physical activity

Model 3: further adjusted for hypertension

## Discussion

Our study revealed that severe periodontitis and moderate periodontitis were associated with stroke, but mild periodontitis was not associated with stroke. Our findings may suggest that preventing mild periodontitis from progressing to moderate and severe periodontitis could reduce risk of stroke. The risk of stroke in participants with severe periodontitis and moderate periodontitis was 2.55 times and 1.71times as high as those without periodontitis. Smoking and hypertension may mediate the association between periodontitis and stroke. The risk of stroke gradually increased with the aggravation of periodontitis.

Multiple approaches were used to determine the severity of periodontitis in previous studies. The combination of International Classification of Diseases codes and the recommendation of dentists were used to classify periodontal disease in a retrospective cohort study using the National Health Insurance Service-National Health Screening Cohort in Korea [19]. Presence or absence of periodontal pockets was assessed to classify periodontal disease in a study comprised of 9962 adults who participated in the NHANES I [20]. PPD and CAL of six sites per tooth were measured to identify the severity of periodontitis in our study. Compared with previous studies, the combination of PPD and CAL measurement was more precise to classify periodontitis to ensure the reliability of the classification in our study. To our knowledge, the definition of periodontitis from the 2012 Centers for Disease Control and Prevention/American Academy of Periodontology case definitions is used for the first time to explore the association between periodontitis and stroke.

Previous studies investigated the association between periodontitis and systemic diseases, such as diabetes and hypertension. Periodontitis was positively associated with risk factors of stroke. A cross-sectional study showed there was a significantly greater prevalence of severe periodontitis in participants with poorly controlled diabetes than those without diabetes in the NHANES III, which showed that periodontitis was associated with diabetes [21]. In two independent large surveys of the US (n = 3460) and Korean (n = 4539), participants with periodontitis had a higher risk of hypertension and systolic blood pressure greater than 140 mmHg, which indicated a connection between periodontitis and hypertension [22]. In addition, atrial fibrillation is also a major cause of stroke. Previous studies showed that atrial fibrillation might play an important role in the association between periodontitis and stroke [23]. In the Dental Atherosclerosis Risk in Communities cohort study, 5958 individuals were included at baseline, among whom 754 developed atrial fibrillation over a 17-year follow-up period. Time-to-event analysis showed that there was a significant association between severe periodontal disease and atrial fibrillation (HR = 1.31, 95% CI 1.06–1.62) [23]. These studies showed that periodontitis was positively associated with risk factors of stroke, which theoretically supported our findings of the association between periodontitis and stroke.

Increasing evidence indicated that periodontal disease was associated with stroke. A case–control study involving 303 participants with ischemic stroke or transient ischemic attack and 300 representative participants showed that a mean CAL > 6 mm had a 7.4 times higher risk of cerebral ischemia than subjects without periodontitis or gingivitis (95% CI 1.55–15.3), which indicated that periodontitis has a significant impact on cerebral ischemia [24]. The Dental Atherosclerosis Risk in Communities cohort study including a total of 6,736 subjects, 299 participants occurred ischemic strokes over a 15-year follow-up period, reporting that periodontal disease had a higher risk for incident ischemic stroke after adjusting for race/center, age, sex, BMI, hypertension, diabetes mellitus, low-density lipoprotein level, smoking, pack years and education. [10] A retrospective cohort study retrieved from the Taiwanese NHI Research Database including 719,436 subjects showed that after adjustment for confounders, the dental prophylaxis and intensive treatment groups in the periodontal disease group had a significant lower HR for stroke than the non-periodontal disease group (HR = 0.78 and 0.95; 95% CI 0.75–0.81 and 0.91–0.99, respectively) [25]. Our study explores the relationship in a larger sample size and multiple races/ethnicities. Compared with previous studies, we used a more precise measurement considering both PPD and CAL to classify the degree of periodontitis according to the 2012 Centers for Disease Control and Prevention/American Academy of Periodontology case definitions.

We speculated that there were several possible mechanisms underlying the association of stroke with periodontitis. Periodontal bacteria establish themselves within subgingival periodontal pockets of patients susceptible to periodontitis. These bacteria promote the progression of periodontitis by pathologic alteration of the subgingival microbial environment and modulation the hosts inflammatory response. Periodontal bacteria mainly included Porphyromonas gingivalis, Tannerella forsythia and Treponema denticola [26]. Lipopolysaccharide (LPS) and other virulence factors from these periodontal pathogens stimulated host macrophages and inflammatory cells to release some pro-inflammatory cytokines infiltrating the local tissues [26–28]. These pro-inflammatory cytokines included interleukin (IL)-6, IL-1β, tumor necrosis factor (TNF)-a, as well as matrix metalloproteinases [26–28]. As the severity of periodontitis increases, so does the concentration of circulating inflammatory mediators which gradually accumulate to form a systemic inflammatory response [29, 30]. The long-term activity of specific bacteria in periodontitis stimulated the secretion of C-reactive protein [31]. C-reactive protein contributed to atherosclerosis progression by promoting platelet activation and modulating inflammatory response and immune response [32]. It was known that atherosclerosis contributes to the development of stroke.

Lipid metabolism can also be included in mechanisms underlying the association of stroke with periodontitis. LPS induced monocytes to release IL-1β and TNF-α, which could regulate lipid metabolism through alteration in hypothalamic–pituitary–adrenal axis, inducement to relevant cytokine production, increase in hormone plasma concentration, and change in hemodynamics/amino acid utilization [33, 34]. The LPS of Porphyromonas gingivalis could change the gene expression of macrophages, resulting in upregulation of the genes encoding cholesterol production and foam cell formation [35]. Lipid metabolism provided an another important clue leading to the onset of stroke, which was able to partly explain the association between periodontitis and stroke [36].

Smoking mediates the association between periodontitis and stroke as we found that the association was alleviated after adjusting for smoking in our study. Smoking is an important modifiable risk factor for chronic periodontitis. The underlying mechanisms included the ability of the periodontium to heal, the composition of the microbiota, and the regulation of immune responses [37]. Meanwhile, smoking is also a significant risk factor for stroke [16]. A meta-analysis showed that smokers had an overall increased risk of stroke compared with nonsmokers [38]. As smoking was associated with both stroke and periodontitis, smoking control contributes to prevent both stroke and periodontitis.

Hypertension also mediates the association between periodontitis and stroke as we found that the association was alleviated after adjusting for hypertension in our study. A cross-sectional study involving 5934 participants from Hamburg City showed that the odds of hypertension increased significantly along with periodontitis severity [39]. Endothelial damage and systemic inflammation were main causes of increasing blood pressure in patients suffering from periodontitis [40, 41]. Hence, blood pressure measurement is of importance to stroke and periodontitis.

There were several limitations in this study. Firstly, due to the nature of cross-sectional study, it inevitably avoids determining a temporal association between periodontitis and stroke. However, it is more reasonable to understand that periodontitis contributes to the development of stroke in our study. Secondly, ischemic stroke and hemorrhagic stroke are not differentiated in survey data. Further studies are expected to be conducted to explore the association of periodontitis with stroke subtypes. Thirdly, we are unable to obtain information on the participants' control status of systemic diseases from the dataset, but these systemic diseases are generally in a long-term stable state. Thus, the data are able to represent participants’ control status of systemic diseases. Fourthly, there is a lack of NHANES validation data on self-reported stroke diagnosis. However, the previous stroke epidemiological study reported that the estimated sensitivity of self-reported stroke was approximately 80% and the specificity was 99% [42]. The diagnosis of stroke was based not only on self-reporting by the participants, but also on assessment by doctors or other health professionals in our study.

## Conclusion

In this nationally representative study, we found that severe periodontitis and moderate periodontitis were associated with stroke. The risk of stroke in participants with severe periodontitis and moderate periodontitis was 2.55 times and 1.71 times as high as those without periodontitis. Our findings may suggest that preventing mild periodontitis from developing into moderate or severe periodontitis may further reduce risk of stroke. Dental health management may be of benefit to stroke prevention.

### Supplementary Information

**Additional file 1: Figure S1.** Flowchart of the study population. **Table S1.** Classification of Periodontitis.

## Data Availability

The National Health and Nutrition Examination Survey dataset is publicly available at the National Center for Health Statistics of the Center for Disease Control and Prevention (https://www.cdc.gov/nchs/nhanes/index.htm).
