# Supplementary material for: Periodontitis is associated with stroke
Source: J Transl Med. 2023 Oct 6;21:697. doi: 10.1186/s12967-023-04545-1 (PMC10559622; doi:10.1186/s12967-023-04545-1)
Supplement: Supplementary file 1 — Additional file 1: Figure S1. Flowchart of the study population. Table S1. Classification of Periodontitis. [file 12967_2023_4545_MOESM1_ESM.docx]

Figure S1. Flowchart of the study population.

NHANES 2009-2014

(n=30,468)

Complete information about periodontitis and stroke

(n=7,042)

No complete information about periodontitis and stroke

(n=23,426)

Missing data on confounders in the analysis models (n=582)

Final study sample

(n=6,460)

Representing 92,856,028 American citizens

Stratified, multi-stage probability sampling designs after survey-weighting

Table S1. Classification of Periodontitis

| Classification | CAL |  | | | PPD | |
| --- | --- | --- | --- | --- | --- | --- |
| No periodontitis | No evidence of mild, moderate or severe periodontitis | | | | | |
| Mild periodontitis | ≥2 interproximal sites with CAL ≥ 3 mm | | | and | | ≥2 interproximal sites with PPD ≥ 4 mm (not on the same tooth) or one site with PPD ≥ 5 mm |
| Moderate periodontitis | ≥ 2 interproximal sites with CAL ≥ 4 mm (not on the same tooth) | | | or | | ≥2 interproximal sites with PPD ≥ 5 mm (not on the same tooth) |
| Severe periodontitis | ≥2 interproximal sites with CAL ≥ 6 mm (not on the same tooth) | | and | | | ≥1 interproximal site with PPD ≥ 5 mm |

CAL: clinical attachment level; PPD: probing pocket depth
